# Role of body mass index in the relationship between adverse childhood experiences, resilience, and mental health: a multivariate analysis

**DOI:** 10.1186/s12888-023-04869-8

**Published:** 2023-06-23

**Authors:** Yi Zhang, Yonghan Li, Tian Jiang, Qiu Zhang

**Affiliations:** 1 Department of Endocrinology, The First Affiliated Hospital of Anhui Medical University, Hefei, 230022 Anhui China; 2 Department of Maternal, Child and Adolescent Health, School of Public Health, Anhui Medical University, No 81 Meishan Road, Hefei, 230032 Anhui China

**Keywords:** Body Mass Index, Mental health, Resilience, Adverse childhood experiences, Adolescents

## Abstract

**Objectives:**

Depression among adolescents is a global concern. Adverse childhood experiences (ACEs) have been correlated with negative physical and mental health such as obesity and depression; however, increasing evidence has suggested that their correlation might be moderated by BMI and resilience. In this study, we aim to explore (1) whether resilience moderate the risk of mental health by ACEs; (2) whether BMI is a moderator of this relationship.

**Study Design:**

Adolescents were obtained from 4 grade college students by a multi-stage convenience sampling method in the period of May to Jun, 2022.

**Methods:**

We use the Connor-Davidson Resilience scale, Depression, Anxiety and Stress Scale-21 Item (DASS-21) questionnaires to measure the ACEs, BMI, resilience and mental health. The primary exposure was ACEs and the primary outcome was mental health; while resilience and BMI were moderators. Multivariable linear regression model was used to establish the relationship of ACEs, resilience and BMI against mental health status. Moderate analysis was employed by PROCESS method to explore the relationship between these variables.

**Results:**

A total of 3600 individuals were initially enrolled, after excluding 22 with invalid questionnaires, 3578 adolescents were finally included. The mean age was (20.53 ± 1.65) years old. After adjusted for covariates, multivariable linear regression suggest that the high level ACEs (, β =0.58, , 95%CI:0.54,0.62, P < 0.01), resilience (, β=-0.27, 95%CI: , 95%CI: -0.28,-0.26, P < 0.01) were associated with higher depression symptoms, and BMI (, β =0.073, 95%CI: 0.002–0.15, P < 0.05) was associated with higher depression symptoms. There is also the interaction between resilience, ACEs and mental health (depression, anxiety and stress symptoms). In the relationship between ACEs and mental health, resilience and BMI played a moderator role.

**Conclusions:**

The moderate analysis also provided further evidence of a link between resilience, ACEs, BMI and mental health. The findings shed new light on potential mechanisms between ACEs and mental health, including the effects of the co-interaction of resilience and BMI, adding to previous literature. ACEs may be a profound variable to measure adolescents’ psychosocial environment to influence mental health, and resilience moderate this effect and is also moderated by BMI.

## Introduction

Mental health, including depressive and anxiety symptoms, is a complex condition affected by multiple factors. Previous studies have concluded that the lifetime prevalence ranges of major depressive disorder (MDD) coverd from 2 to 21% [1]. In 2008, the WHO classifies MDD as the third leading cause of the global burden of disease, and expects it to be number one by 2030 [2]. Depression is the second most common mental disorder among adolescents, more people were defined as having depressive symptoms and is the focus of attention in mental health (public health). The overall pooled crude prevalence of depression or depressive symptoms was 27.2% (37,933/122,356 individuals; 95%CI = 24.7-29.9%, I^2^ = 98.9%) [3]. Another study also reported that the global prevalence of increased self-reported depressive symptoms from 2001 to 2020 was 34% (95% CI:0.30–0.38) [4]. The prevalence of anxiety ranges from 13 to 67%, with an overall pooled prevalence of 39% [5]. Adolescent mental health is a complex problem facing all countries today, with the development and needs of society and the guidance of sustainable development goals, mental health education, and mental health services receiving increasing attention.

These stark figures highlight the urgent need to improve prevention and treatment and the rising global demand to curb mental health conditions. However, progress has been hampered by the lack of a reliable way to predict various psychological problems and an insufficient understanding of their biological causes. Although most research and public health researchers believe that adolescent depression is due to individual, family, or school reasons, these factors cannot fully explain the persistently high and unnecessary incidence of health risk behaviors in modern society. Therefore, it is necessary to identify factors contributing to mental health and achieve this premise. It should be based on a theory applicable to multiple behaviors.

The empirical study examined that adverse childhood experiences (ACEs) were associated with depression [6, 7]. The buffering model of social support proposes that sufficient support can mitigate the impact of stressful events, thereby promoting emotional and behavioral adjustment and serving as an essential mediator between ACEs and mental health outcomes [8–10]. Similarly, resilience has been found to play a similar buffering role in the relationship between ACEs and mental health outcomes [11, 12], underscoring its importance in promoting adaptive coping in the face of adversity [12]. Research on emerging psychopathology should also focus on factors influencing risk and resilience [13]. The phenomenological variant of ecosystem theory (PVEST), a theoretical framework for explaining the effects of resilience, can be used to address this link [14]. In addition, PVEST also focuses on the ecosystem of individual growth. This theory emphasizes the process of individual coping and adaptation in terms of resource acquisition, coping and adaptation, which coincides with the idea of ACEs and can directly affect the behaviors and health related to individuals. Based on this theory, we propose that many factors influence psychological problems in different environmental backgrounds (unpublished data from our research group). This study constructed the overall living environment of adolescents through the variable of ACEs according to PVEST to explore their role in adolescent mental health problems. Numerous studies have been reported on the factors that influence the development of mental disorders. Despite this, relatively little is known about the factors that contribute to positive development (resilience factors) when exposed to atypically high stress levels or adversity [15].

Other factors are also involved in this relationship. These problems can occur in different cultures and at any age, and also have a high prevalence in the general population. Mcgue and Bouchard point out that for most psychological and physiological variables, age and sex have a significant influence [16]. In addition, obesity is increasingly being linked to, and may even be a cause of, mental illness [17], but how changes in BMI co-moderate resilience ACEs research on mental health not yet available. In addition, Deng et al. used the UK Millennium Cohort Study to investigate the relationship between adverse childhood experiences (ACEs) in early childhood (9 months and 3 years) and the obesity trajectory of children/adolescents aged 5 to 17 years, and found some correlation [18]. There is strong evidence that obese female children have much higher rates of depression than female children of normal weight and that this risk persists into adulthood [17]. How the effect of obesity on mental health interacts with psychological resilience and ACEs is also worth exploring. However, the mechanism underlying the association between ACEs, BMI risk, and resilience to adverse psychological factors remains unclear.

We aimed to achieve multi-dimensional screening of depression and anxiety symptoms in the annual physical examination and the high incidence period of depression in critical groups of adolescents to promote the healthy growth of adolescents’ mental health. In combining the above multi-dimensional research results, a systematic and comprehensive screening and prevention program for adolescent mental health was proposed, providing a strong basis for relevant guidelines or the establishment of expert consensus on the prevention technology of adolescent mental health. These studies confirm the moderating role of resilience in the relationship between ACEs and mental health. Literature search shows that there are few relevant studies, even less studies on Chinese college students. Based on the above results, the following hypothesis was proposed in this study with Chinese college students as the target population: resilience and BMI are the moderating variables of the relationship between ACEs and the mental health of college students.

## Methods

### Study design

This cross-sectional study used an extensive survey procedure to assess the sociodemographic and psychosocial environmental factors that contribute to the mental health and health risk behaviors of the general population [19, 20]. The present study was designed and reported according to the Strengthening the Reporting of Observational Studies in Epidemiology (STROBE) checklist. Before the investigation, all on-site investigators were trained, and the working standards were unified.

### Settings

A cross-sectional study design was used to conduct nationwide sample surveys in Anhui Province, China. We took into account both the sampling method and the partnership. We randomly selected 2 schools according to the geographical distribution and the degree of school cooperation [21–23]. The study participants were college students sampled from May 2022 to June 2022 in Anhui Province using multi-stage convenience sampling method. The study subjects were class-based, and students were required to complete the questionnaire anonymously on the spot during non-class hours, according to the principle of informed consent. The teacher maintained order in the classroom for approximately 20–30 min, and the quality control personnel answered the questions of the survey subjects on the spot and were responsible for collecting and reviewing the questionnaire. The design and data collection procedures were approved by the Ethics Committee of Anhui Medical University. Further, written informed consent was obtained from the parents or guardians of all students. All methods were performed per relevant guidelines and regulations.

### Inclusion criteria

Participants: (1) obtained informed consent from the participants and their guardians; (2) were college students [aged 15–26 years]; (3) had no history of mental illness; and (4) were attending the chosen school.

### Exclusion criteria

Participants: (1) Informed consent was not obtained; (2) age between 15 and 26 years; (3) failure to submit the questionnaire; and (4) not college students from the chosen school.

### Exposure

#### Adverse childhood experiences

The adverse childhood experiences scale has 22 items [24, 25], which include five dimensions: emotional abuse, physical abuse, sexual abuse, emotional neglect, and physical neglect, with five items in each dimension, totaling 22 items. A 5-level score of “never, rarely, sometimes, often, and always” was applied to each item, and the items whose results were “0 = never”, “1 = rarely”, “2 = sometimes”, “3 = often,“ and “4 = always” were defined as correspondent items. Positive items with more than one item in each dimension were defined as having experienced abuse. The internal consistency coefficient of the questionnaire adopted was 0.75.

#### Resilience

The Chinese version of the Connor-Davidson resilience scale compiled by Kathryn et al. was used to evaluate the mental resilience of college students [26, 27]. The scale comprises three dimensions (tenacity, self-improvement, and optimism) and contains 25 items. Each item adopts Likert 5-level scoring method(1–5 points), with a total score of 100 points. Higher scores indicate higher levels of mental resilience.

### Outcome

#### Mental health

The Depression, Anxiety and Stress Scale-21 Item (DASS-21) used in this study measures the mental health status of students and is an effective and reliable psychological tool that has been shown to accurately measure depression, anxiety and stress [28]. The DASS-21 is a self-reported survey with three subscales: depression (DASS-21D), anxiety (DASS-21 A), and stress (DASS-21 S), each with seven items. Each item on the questionnaire provided a statement and four response options to assess the severity of the participant’s experience, on a scale of 0 (does not apply to me at all) to 3 (applies to me very much). The DASS-21 can measure all three parameters over the past week. The intensity of any of the three conditions was determined by adding scores for seven items to the subscale, ranging from 0 to 21 for each, with higher scores indicating higher severity [28].

### Covariates

Sex, age, parental education level, residential area, only child status, family economic status, number of friends, and academic records were included as covariates.

### Statistics analysis

Continuous variables were described using mean and standard deviation (SD) and one-way analysis of variance. Categorical variables were described using frequencies and percentages using the Chi-square test. Multivariate logistic regression was conducted to evaluate the relationships among life events, chronotype, mental health, and health risk behaviors, presented as adjusted odds ratios (aORs) with 95% confidence intervals (CIs). Moderate mediation analysis was employed using the PROCESS method to explore the relationships among ACEs, BMI, resilience and mental health [29]. The bootstrap method was used to re-sample 1000 samples, and 95% CI were calculated. All analyses were conducted using SPSS Windows software version 23.0. After preliminary data sorting, missing data were processed using SPSS. Generally, the missing data rate in this study was meager, with the missing rate for each item being less than 1%. Therefore, a multiple imputation method was adopted in SPSS to process missing data at the project level.

## Results

### General demographic statistics

Among the 3578 students in the questionnaire included in the analysis, 1833 were male students, and 1745 were female students; the age was (20.53 ± 1.65) years old; 1665 (46.5%) lived in rural areas, 783 (21.9%) lived in towns, and 1130 (31.6%) live in cities. There were 1180 only children (33.0%) and 2398 non-only children (67.0%). Among them, 2100 (58.7%) had junior high school education or below, and 1350 (37.7%) had senior high school education or above. A total of 2457 (68.7%) of mothers had junior middle school education or below, and 968 (27.1%) had senior high school education or above. Among them, 1103 (30.8%) were classified as poor, 2242 (62.7%) as moderate, and 233 (6.5%) as good. A total of 1272 (35.6%) had less than two friends, 1730 (48.4%) had three to five friends, and 576 (16.1%) had more than six friends. The prevalence rates of depression and anxiety symptoms were 37.5% and 11.6%, respectively. The demographic characteristics and mental health assessment results are presented in Tables 1 and 2.

**Table 1:** The distribution of demographic characteristics on depression

Variables	Total (N, %)	No (n,%)	Have (n,%)	*t/χ*^2^ value
Age		20.54 ± 1.32	20.51 ± 2.08	0.52
Gender				69.29**
Male	1833	1025(55.9)	808(44.1)	
Female	1745	1211(69.4)	534(30.6)	
Residential areas				2.95
Country	1665	1018(61.1)	647(38.9)	
Town	783	491(62.7)	292(37.3)	
Urban	1130	727(64.3)	403(35.7)	
Only child				1.64
Yes	11,280	720(61.0)	460(39.0)	
No	2399	1516(63.2)	882(36.8)	
Father’s education				26.74**
Not clear	128	58(45.3)	70(54.7)	
Below primary	318	191(60.1)	127(39.9)	
Primary	427	282(66.00	145(34.0)	
Junior high	1355	862(63.6)	493(36.4)	
High school or technical secondary school	732	434(59.9)	298(40.7)	
Junior college or above	618	409(66.2)	209(33.8)	
Mother’s education				24.36**
Not clear	153	67(43.8)	86(56.2)	
Below primary	729	456(62.6)	273(37.4)	
Primary	628	401(63.9)	227(36.1)	
Junior high	1100	699(63.5)	401(36.5)	
High school or technical secondary school	552	346(62.7)	206(37.3)	
Junior college or above	416	267(64.2)	149(35.8)	
Family economic conditions				35.88**
Bad	212	98(46.2)	114(53.8)	
Worse	891	538(60.4)	353(39.6)	
Medium	2242	1462(65.2)	780(34.8)	
Better	207	126(60.9)	81(39.1)	
Good	26	12(46.2)	14(53.8)	
Friends number				84.12**
No	85	27(31.8)	58(68.2)	
1–2	1187	656(55.3)	531(44.7)	
3–5	1730	1160(67.1)	570(32.9)	
6 or more	576	393(68.2)	183(31.8)	

*P<0.05, **P<0.01

**Table 2:** The distribution of demographic characteristics on anxiety

Variables	Total (N, %)	No (n,%)	Have (n,%)	*t/χ*^2^ value
Age		20.53 ± 1.57	20.53 ± 2.11	0.07
Gender				8.52**
Male	1833	1593(86.9)	240(13.1)	
Female	1745	1571(90.0)	174(10.0)	
Residential areas				5.21
Country	1665	1455(87.4)	210(12.6)	
Town	783	690(88.1)	93(11.9)	
Urban	1130	1019(90.2)	111(9.8)	
Only child				0.38
Yes		1049(88.9)	131(11.1)	
No		2115(88.20	283(11.80	
Father’s education				4.30
Not clear	128	110(85.9)	18(14.1)	
Below primary	318	275(86.50	43(13.5)	
Primary	427	378(88.50	49(11.5)	
Junior high	1355	1214(89.6)	141(10.4)	
High school or technical secondary school	732	641(87.6)	91(12.4)	
Junior college or above	618	546(88.3)	72(11.7)	
Mother’s education				11.43*
Not clear	153	127(83.0)	26(17.0)	
Below primary	729	634(87.0)	95(13.0)	
Primary	628	554(88.2)	74(11.8)	
Junior high	1100	984(89.5)	116(10.5)	
High school or technical secondary school	552	484(87.7)	68(12.3)	
Junior college or above	416	381(91.6)	35(8.4)	
Family economic conditions				41.17**
Bad	212	160(75.5)	52(24.5)	
Worse	891	781(87.7)	110(12.3)	
Medium	2242	2014(89.8)	228(10.2)	
Better	207	184(88.9)	23(11.1)	
Good	26	25(96.2)	1(3.8)	
Friends number				42.01**
No	85	63(74.1)	22(25.9)	
1–2	1187	1017(85.7)	170(14.3)	
3–5	1730	1545(89.3)	185(10.7)	
6 or more	576	539(93.6)	37(6.4)	

*P<0.05, **P<0.01

### Association between independent variables and adolescent psychological and behavioral problems

In Table 3, there was a dose‒response relationship between different ACEs and depression (emotional abuse: β = 1.13, physical abuse: β = 1.21, sexual abuse: β = 1.28, emotional neglect: β = 0.97, physical neglect: β = 0.16, ACEs: β = 0.62). After controlling for covariates, the relationships were statistically significant. There was also a negative dose–response relationship between resilience and depression (β=-0.28), and after controlling for covariates, this relationship was significant (β=-0.27). Similar results are presented in Tables 4 and 5.

**Table 3:** The multilevel linear regression between independent variables and depression

	Depression						
Emotional abuse	R^2^	β	t	p	F	LLCI	ULCI
Model 1	0.03	1.19	10.19	<0.01	109.97	0.97	1.42
Model 2	0.07	1.14	10.15	<0.01	31.61	0.92	1.36
Physical abuse							
Model 1	0.017	1.21	7.76	<0.01	60.18	0.91	1.52
Model 2	0.06	1.06	6.88	<0.01	25.11	0.76	1.36
Sexual abuse							
Model 1	0.01	1.28	5.96	<0.01	35.56	0.86	1.70
Model 2	0.055	1.12	5.31	<0.01	22.89	0.70	1.53
Emotional neglect							
Model 1	0.17	0.97	27.18	<0.01	738.49	0.90	1.04
Model 2	0.19	0.91	25.11	<0.01	93.14	0.84	0.98
Physical neglect							
Model 1	0.16	1.55	25.80	<0.01	665.79	1.43	1.67
Model 2	0.17	1.45	23.46	<0.01	83.78	1.33	1.57
ACEs							
Model 1	0.20	0.62	29.89	<0.01	893.37	0.58	0.66
Model 2	0.22	0.58	27.60	<0.01	108.38	0.54	0.62
BMI							
Model 1	0.004	0.14	3.76	<0.01	14.10	0.065	0.21
Model 2	0.048	0.073	2.01	<0.05	22.39	0.002	0.15
Resilience							
Model 1	0.35	-0.28	-44.13	<0.01	1947.42	-0.29	-0.27
Model 2	0.36	-0.27	-42.05	<0.01	253.74	-0.28	-0.26

Model 1: crude model; Model 2: Controlled for parent educational level, gender, economic level, whether only child, friend numbers, residential areas and age. ACE, adverse childhood experience; BMI, body mass index

**Table 4:** The multilevel linear regression between independent variables and anxiety

	Anxiety						
Emotional abuse	R^2^	β	t	p	F	LLCI	ULCI
Model 1	0.05	1.03	12.91	<0.01	166.65	0.88	1.19
Model 2	0.08	0.99	12.53	<0.01	35.29	0.84	1.15
Physical abuse							
Model 1	0.02	0.96	8.67	<0.01	75.10	0.74	1.17
Model 2	0.058	0.86	7.91	<0.01	24.35	0.65	1.08
Sexual abuse							
Model 1	0.02	1.38	9.14	<0.01	83.57	1.08	1.67
Model 2	0.06	1.28	8.64	<0.01	25.75	0.99	1.57
Emotional neglect							
Model 1	0.11	0.55	21.31	<0.01	453.99	0.50	0.61
Model 2	0.13	0.51	19.34	<0.01	60.43	0.46	0.56
Physical neglect							
Model 1	0.11	0.93	21.35	<0.01	455.94	0.85	1.02
Model 2	0.13	0.86	19.19	<0.01	59.78	0.77	0.95
ACEs							
Model 1	0.16	0.39	25.74	<0.01	662.30	0.36	0.42
Model 2	0.17	0.36	23.60	<0.01	81.64	0.33	0.39
BMI							
Model 1	0.003	0.086	3.33	<0.01	11.08	0.035	0.14
Model 2	0.04	0.045	1.76	0.079	19.59	-0.005	0.096
Resilience							
Model 1	0.14	-0.13	-24.50	<0.01	600.33	-0.14	-0.12
Model 2	0.16	-0.12	-22.48	<0.01	85.08	-0.13	-0.11

Model 1: crude model; Model 2: Controlled for parent educational level, gender, economic level, whether only child, friend numbers, residential areas and age. ACE, adverse childhood experience; BMI, body mass index

**Table 5:** The multilevel linear regression between independent variables and pressure

	DASS-pressure						
Emotional abuse	R^2^	β	t	p	F	LLCI	ULCI
Model 1	0.09	0.57	18.53	<0.01	343.25	0.51	0.63
Model 2	0.11	0.54	17.59	<0.01	49.78	0.48	0.60
Physical abuse							
Model 1	0.02	0.37	8.38	<0.01	70.15	0.28	0.45
Model 2	0.05	0.35	8.08	<0.01	21.68	0.27	0.44
Sexual abuse							
Model 1	0.02	0.50	8.30	<0.01	68.86	0.38	0.61
Model 2	0.05	0.47	7.95	<0.01	21.44	0.35	0.59
Emotional neglect							
Model 1	0.006	0.05	4.48	<0.01	20.05	0.03	0.07
Model 2	0.04	0.03	3.01	<0.01	15.20	0.01	0.06
Physical neglect							
Model 1	0.004	0.07	3.76	<0.01	14.10	0.03	0.11
Model 2	0.04	0.05	2.43	<0.05	14.84	0.01	0.08
ACEs							
Model 1	0.03	0.06	93.89	<0.01	9.69	0.05	0.07
Model 2	0.05	0.05	8.22	<0.01	21.94	0.04	0.07
BMI							
Model 1	0.001	0.02	2.29	<0.05	5.25	0.003	0.04
Model 2	0.04	0.02	2.06	<0.05	14.65	0.001	0.04
Resilience							
Model 1	0.03	-0.02	-9.65	<0.01	93.16	-0.03	-0.02
Model 2	0.05	-0.02	-7.97	<0.01	21.47	-0.02	-0.01

Model 1: crude model; Model 2: Controlled for parent educational level, gender, economic level, whether only child, friend numbers, residential areas and age. ACE, adverse childhood experience; BMI, body mass index

### Impact of the moderation analysis between emotional abuse and resilience, Body Mass Index on adolescent mental health

Moderation analyses were conducted for emotional abuse, resilience, BMI, and mental health. Table 6 presents the results. First, emotional abuse did not correlate with the severity of depression (P > 0.05). Resilience was associated with depression (P < 0.01), emotional abuse× resilience did not significantly correlate the severity of depression, emotional abuse× BMI did not significantly correlate the severity of depression, and resilience× BMI was correlated with depression. There was no three-way interaction effect between emotional abuse, resilience and BMI (P > 0.05). After controlling for education level, marital status, total annual household income, ethnicity, and sex, the results were similar.

**Table 6:** Association between life events and resilience, chronotype and depression in adolescent

Variables	DASS-depression					
	*coeff*	*SE*	*t* value	*P* value	LLCI	ULCI
Emotional abuse	1.42	0.27	5.19	<0.01	0.88	1.95
Resilience	0.05	0.02	1.97	<0.05	0.0002	0.09
BMI	0.19	0.06	3.02	<0.01	0.07	0.32
Int_1	-0.015	0.004	-3.55	<0.01	-0.02	-0.007
Int_2	-0.04	0.01	-3.75	<0.01	-0.06	-0.02
Int_3	-0.003	0.001	-2.98	<0.01	-0.005	-0.001
Int_4	0.0007	0.0002	3.79	<0.01	0.0003	0.001

Int 1: Emotional abuse × resilience; Int 2: Emotional abuse × BMI; Int 3: resilience × BMI; Int 4: Emotional abuse × resilience × BMI. BMI, body mass index

Among the anxiety results, there was a three-way interaction effect between emotional abuse and resilience, BMI, and anxiety (P < 0.05). After controlling for education level, marital status, total annual household income, ethnicity, and sex, the results were similar. Table 7 presents the results. Among the pressure results, there was a three-way interaction effect between emotional abuse and resilience, BMI, and anxiety (P < 0.01). After controlling for education level, marital status, total annual household income, ethnicity and sex, the results were similar. Table 8 presents the results of the study.

**Table 7:** Association between life events and resilience, chronotype and anxiety in adolescent

Variables	DASS-anxiety					
	*coeff*	*SE*	*t* value	*P* value	LLCI	ULCI
Emotional abuse	0.88	0.27	3.21	<0.01	0.34	1.41
Resilience	0.007	0.02	0.29	>0.05	-0.04	0.05
BMI	0.12	0.06	1.81	>0.05	-0.009	0.24
Int_1	-0.007	0.004	-1.71	>0.05	-0.016	0.001
Int_2	-0.028	0.01	-2.51	<0.05	-0.05	-0.006
Int_3	-0.002	0.001	-1.62	>0.05	-0.004	0.0003
Int_4	0.0004	0.0002	2.42	<0.05	0.0001	0.0008

Int 1: Emotional abuse × resilience; Int 2: Emotional abuse × BMI; Int 3: resilience × BMI; Int 4: Emotional abuse × resilience × BMI. BMI, body mass index

**Table 8:** Association between life events and resilience, chronotype and pressure in adolescent

Variables	DASS-pressure					
	*coeff*	*SE*	*t* value	*P* value	LLCI	ULCI
Emotional abuse	1.21	0.31	3.88	<0.01	0.60	1.82
Resilience	0.02	0.03	0.75	>0.05	-0.03	0.07
BMI	0.15	0.07	2.03	<0.05	0.005	0.29
Int_1	-0.01	0.005	-2.26	<0.05	-0.02	-0.001
Int_2	-0.04	0.01	-2.92	<0.01	-0.06	-0.01
Int_3	-0.002	0.001	-1.90	0.054	-0.005	0.0001
Int_4	0.0006	0.0002	2.85	<0.01	0.0002	0.001

Int 1: Emotional abuse × resilience; Int 2: Emotional abuse × BMI; Int 3: resilience × BMI; Int 4: Emotional abuse × resilience × BMI. BMI, body mass index

### Impact of the mediate moderation analysis between adverse childhood experiences and resilience, Body Mass Index on adolescent mental health

Moderation analyses were performed with clustering of ACEs and resilience, BMI, and mental health. The results are presented in Tables 9, 10 and 11. First, ACEs did not correlate with the severity of depression (P > 0.05), resilience was associated with depression, and ACEs× resilience did not significantly correlate the severity of depression (P > 0.05). ACEs×BMI did not significantly correlate depression severity, and resilience×BMI did not correlate with depression. There was no three-way interaction effect between the ACEs, resilience, and BMI (P > 0.05). These results bring into correspondence with previous results after controlling for covariates.

**Table 9:** Association between life events and resilience, chronotype and depression in adolescent

Variables	DASS-depression					
	*coeff*	*SE*	*t* value	*P* value	LLCI	ULCI
ACEs	-0.05	0.06	-0.79	>0.05	-0.17	0.07
Resilience	-0.05	0.03	-1.58	>0.05	-0.11	0.01
BMI	0.09	0.11	0.87	>0.05	-0.12	0.30
Int_1	0.0003	0.0008	0.39	>0.05	-0.001	0.002
Int_2	-0.004	0.0025	-1.45	>0.05	-0.008	0.001
Int_3	-0.002	0.001	-1.57	>0.05	-0.005	0.0005
Int_4	0.0001	0.000	2.46	<0.05	0.000	0.0001

Int 1: ACEs × resilience; Int 2: Emotional abuse × BMI; Int 3: resilience × BMI; Int 4: Emotional abuse × resilience × BMI. ACE, adverse childhood experience; BMI, body mass index

**Table 10:** Association between life events and resilience, chronotype and anxiety in adolescent

Variables	DASS-anxiety					
	*coeff*	*SE*	*t* value	*P* value	LLCI	ULCI
ACEs	0.013	0.05	0.24	>0.05	-0.09	0.12
Resilience	-0.02	0.028	-0.73	>0.05	-0.07	0.03
BMI	0.20	0.09	2.09	<0.05	0.01	0.38
Int_1	-0.0002	0.0007	-0.30	>0.05	-0.002	0.001
Int_2	-0.006	0.002	-2.56	<0.05	-0.01	-0.001
Int_3	-0.003	0.001	-2.53	<0.05	-0.005	-0.0007
Int_4	0.0001	0.000	3.21	<0.01	0.0000	0.0002

Int 1: ACEs × resilience; Int 2: Emotional abuse × BMI; Int 3: resilience × BMI; Int 4: Emotional abuse × resilience × BMI. ACE, adverse childhood experience; BMI, body mass index

**Table 11:** Association between life events and resilience, chronotype and pressure in adolescent

Variables	DASS-pressure					
	*coeff*	*SE*	*t* value	*P* value	LLCI	ULCI
ACEs	-0.05	0.06	-0.79	>0.05	-0.17	0.07
Resilience	-0.05	0.03	1.58	>0.05	-0.11	0.01
BMI	0.09	0.11	0.87	>0.05	-0.12	0.30
Int_1	0.0003	0.0008	0.38	>0.05	-0.001	0.002
Int_2	-0.004	0.003	-1.45	>0.05	-0.008	0.001
Int_3	-0.002	0.001	-1.57	>0.05	-0.005	0.0005
Int_4	0.0001	0.000	2.46	<0.05	0.000	0.0001

Int 1: ACEs × resilience; Int 2: Emotional abuse × BMI; Int 3: resilience × BMI; Int 4: Emotional abuse × resilience × BMI. ACE, adverse childhood experience; BMI, body mass index

Among the anxiety results, there was a three-way interaction effect between ACEs and resilience, BMI, and anxiety (P < 0.05). After controlling for education level, marital status, total annual household income, ethnicity and sex, the results were similar. Among the pressure results, there was a three-way interaction effect between ACEs and resilience, BMI, and pressure (P > 0.05). After controlling for education level, marital status, total annual household income, ethnicity and sex, the results were similar.

## Discussion

### Principal findings

The study examined the relationship between ACEs, BMI, resilience and mental health in a sample of current college students. The prevalence rates of depression, anxiety and stress symptoms were 18.6%, 24.5% and 5.8%, respectively. Second, there is an interaction between ACEs, BMI, and mental health, and between ACEs, resilience and mental health. Third, the study also found a relationship between resilience-moderate ACEs and mental health. The results verify the stress buffering effect model of resilience. The stress buffer effect model showed that higher level resilience could cushion the adverse effects of ACEs on emotional problems. Finally, our results also found a three-way interaction between ACEs, BMI, and resilience. Our results support research hypotheses regarding different factors and underlying mechanisms of student mental health. In addition, multiple layers of trauma-informed early childhood interventions are needed to prevent adverse adolescent outcomes associated with ACEs [30].

### The correlation between ACEs and mental health

Similar study demonstrated that 29% of the students were depressed, 27% were anxious, and 24% were stressed; About 67% of students who were anxious were also depressed and 61% of the anxious students were also stressed [31, 32]. Several factors account for this phenomenon [33, 34]. These include daily life stressors and stressors specific to tedious learning environments. Our study had a sex difference in mental health, similar to another study’s findings [35, 36]. ACEs including physical, sexual, or psychological abuse or neglect of a child or children, especially by a parent or other caregiver [24]. Based on social-ecological risk factor research, some studies point out that teenagers with high ACE scores are at a higher risk of psychological, emotional, and behavioral problems. Further, those with lower social-ecological risk and teenagers with medium or high risk in internal and external behavior differences [37]. No single risk factor plays a decisive role in forming an individual’s healthy development, and the effect of intervention targeting only a single risk is significantly reduced. Specifically, for adolescents, an individual risk factor may not threaten mental health development; only when risk factors are cumulative can they significantly impact personal development [14]. Our results demonstrated that cumulative life events were positively related to poor mental health, per ecological system theory. Since the pioneering studies on ecological risk, cumulative risk has gained support in various social and cognitive domains [38, 39]. Based on these findings, we propose that ACEs are also influenced by social ecosystem theory.

### The correlation between ACEs, BMI, resilience and mental health

Our study further explored the relationship between BMI, resilience, ACEs and mental health. First, favorable resilience is associated with lower HRBs and mental health problems [40]. This is consistent with our findings that there is also an association between BMI and mental health. The study also further sheds light on the potential mechanistic relationship of ACEs to mental health and identifies how and what aspects of ACEs can be targeted through therapeutic interventions [41]. Second, a previous study found that favorable resilience was associated with lower mental health [42–44]. Resilience is not only closely related to the mental health of children and adolescents, but also plays a well-regulated role in adults, and should play a more prominent role in real-world research and prevention programs [40, 45]. Our analysis was based on hypotheses derived from the psychopathology between ACEs and mental health [46] and resilience as a potential mediator between adverse childhood experiences and prenatal depression [47, 48]. The impact of ACEs on mental health can be reduced by managing current stressors and improving mental resilience in students [49]. These results provide a theoretical basis for studying the moderating effects of the analyses on the significance of resilience, ACEs, BMI, and mental health. Third, the interaction between high-risk ACEs and obesity correlates with adverse mental health symptoms [50]. It also means that adolescents who are exposed to high-risk social environments, combined with disharmony in life and rest, can lead to physical and mental health [51]. This means that social ecological psychology research is a type of process research that further shows that the specific characteristics of social ecology cause a psychological state. This psychological state can also cause the target’s cognition, emotion, or behavior through some mechanism. These results are similar to those of a previous study [52]. Possible mechanisms were explained “pub hypothesis” [53] and the diathesis-stress model [54] or the comprehensive social-ecological diathesis-stress model [55]. Similar research investigations have been conducted to examine the trajectory of multiple childhood adversity and their relationship to adolescent mental health outcomes, as well as the role of good parenting practices as a buffer [56]. Another study found a moderating effect of resilience and concluded that it may reduce the negative effects of neuroticism and enhance the positive effects of extraversion, agreeableness and conscientiousness on depressive symptoms [40]. This further verifies the association between ACEs, BMI, resilience, and mental health. ACE is a complex etiological marker whose effects appear to vary in terms of the type, timing, and severity of abuse, in addition to a wide range of vulnerability and resilience cofactors [50].

We also explored the moderating effect of resilience on BMI. ACEs are also associated between ACEs and obesity [57, 58]. The possible mechanism is that physiological responses to psychosocial stress (manifested in altered levels of neuroendocrine hormones, the development of malignant stress, and increased allostatic load) are hypothesized to accumulate and interact with each other to further slow metabolic processes. As a result, metabolic related diseases, including cardiovascular, immune and nervous system damage, including the occurrence and development of obesity [58–60]. We tried to tridimensify the correlation between resilience, BMI, and mental health from a social environment perspective (such as the ACEs environment). Identifying factors that promote resilience may be a target of concurrent and retrospective interventions [61]. Our study found that high life event scores were associated with higher resilience and better mental health. Similarly, adolescents who experienced a worse social environment, together with obesity, through decreased resilience, increased adverse mental health. In summary, obesity can moderate mental health problems caused by ACEs mediated by lower resilience. Several researchers have described recent behavioral and neurobiological resilience studies suggesting that adolescence (a period marked by increased plasticity, development of vital neurobiological circuits, and sensitivity to social environments) may be a particularly appropriate time for ELA intervention [62]. Other researchers have also provided insights into the resilience and vulnerability impact of multiple ACEs, highlighting its research value and clinical implications for further understanding of trauma in young people affected by conflict [63]. There is growing evidence that the fundamental mechanisms by which stressors lead to stable changes in behavior include epigenetic changes, which result from interactions with the genome that lead to changes in DNA structure and gene expression [64].

By integrating the diathesis-stress model with a social-ecological framework, we aim to develop a comprehensive approach to understanding mental health that recognizes the complex and dynamic nature of mental health experiences across various settings, such as individuals, families, communities, and schools, and over time. Social-ecological models consider the interconnectedness of the child’s world. Quality-stress models allow an understanding of the complexity of stressors and risk or protective factors that influence participation and intervention in mental and behavioral health [55]. Potential social factors that influence mental health, and the resulting mental health, may be dampened or overridden by obesity. These results corroborate and extend previous research by adopting multiple social environments to portray the whole society of adolescents, together with their physical conditions (in this study, obesity), to explore the moderating role of mental health in resilience and further verify the moderating role of BMI.

It also provides perspectives to investigate further the association between BMI and mental health, resilience, and mental health, in addition to ACEs [65]. Social-ecological risk factors influence adolescent mental health, which may be moderated by obesity and resilience; therefore, we should advocate ameliorating possible ACEs and provide more support at home, school, and other levels.

### Strength and limitation

The strengths of this study are its multilevel design and the large number of college participants. However, this study has some limitations. As a cross-sectional study, it was difficult to observe a causal association between life events and mental health, and longitudinal studies are needed to evaluate the association between variables in the future. All the data used in this study were the results of self-reported questionnaires; therefore, there were questions of subjectivity, validity, and reliability. This study only investigated the results of 1 city, it is not clear how representative this sample may be, and the follow-up survey will be carried out among samples from different regions and cultures across the country. Although this study focused on the association between ACEs and mental health in different fields, it did not cover other fields. Future research should explore the association between ACEs and mental health at various levels across different fields.

## Conclusion

We found that adolescents with high cumulative ACEs experienced high-risk mental health problems. Adolescents with favorable resilience also have low mental health risks. There was also a positive interactive association between cumulative ACEs and obesity, resilience, and mental health. Based on our moderate analysis, we should also consider adolescents’ mental health and improve their obesity status. Resilience is an important aspect of psychopathology in children, adolescents and young adults that should be studied and explored using a multi-systems approach, including individual, social, family and cultural contexts based on the social ecosystem. Resilience is also a dynamic process that changes over time, longitudinal studies prospectively assessing resilience and psychopathology in children and adolescents are required. This study effectively addressed the adverse consequences of adolescent non-communicable diseases (NCDs) by continuously promoting a healthy China, improving the building of the public health system, and strengthening the capacity building of NCDs in preventive medicine and clinical medicine.

## Data Availability

The datasets generated for this study are available on request to the corresponding author.

## List of Abbreviations

ACEs: Adverse childhood experiences
BMI: Body Mass Index
CIs: Confidence intervals
DASS-21: Depression Anxiety and Stress Scale − 21 Items
MDD: Major depressive disorder
NCDs: Non-communicable diseases
PVEST: Phenomenological variant of Ecosystem theory
SAS: Self-rating anxiety scale
WHO: World Health Organization
